# Supplementary material for: The genome and transcriptome of Phalaenopsis yield insights into floral organ development and flowering regulation
Source: PeerJ. 2016 May 12;4:e2017. doi: 10.7717/peerj.2017 (PMC4868593; doi:10.7717/peerj.2017)
Supplement: Supplemental Information 9 [file peerj-04-2017-s009.doc]

**SUPPLEMENTARY INFORMATION APPENDIX**

**The genome and transcriptome of the *Phalaenopsis* yield insights into floral organ development and flowering regulation**

Jian-Zhi Huang^1*^, Chih-Peng Lin^2,4*^, Ting-Chi Cheng^1^, Ya-Wen Huang^1^,Yi-Jung Tsai^1^, Shu-Yun Cheng^1^, Yi-Wen Chen^1^, Chueh-Pai Lee^2^, Wan-Chia Chung^2^, Bill Chia-Han Chang^2,3#^, Shih-Wen Chin^1#^, Chen-Yu Lee^1#^ & Fure-Chyi Chen^1#^

^1^Department of Plant Industry, National Pingtung University of Science and Technology, Pingtung 91201, Taiwan

^2^Yourgene Bioscience, Shu-Lin District, New Taipei City 23863, Taiwan

^3^Faculty of Veterinary Science, The University of Melbourne, Parkville Victoria 3010 Australia

^4^Department of Biotechnology, School of Health Technology, Ming Chuan University, Gui Shan District, Taoyuan 333, Taiwan

^*^These authors contributed equally to this work.

^#^Correspondence should be addressed to B-C.H.C. (bchang@yourgene.com.tw), S.-W.C. (swchin@mail.npust.edu.tw), C.-Y.L. (culee@mail.npust.edu.tw) & F.-C.C. (fchen@mail.npust.edu.tw)

**Table of Contents**

**SI TEXT**........................................................................................................................3

**S1. Genome sequencing and assembly**.......................................................................3

S1.1 Whole genome shotgun sequencing using the Illumina technology.............3

S1.2 Library construction, sequencing and quality control...................................3

S1.3 Estimation of genome size using K-mer........................................................3

S1.4 De novo assembly of the *Phalaenopsis* genome...........................................3

S1.5 Assembly evaluation......................................................................................4

S1.6 GC comparison..............................................................................................4

S.1.7 Identification of differentially expressed transcripts……………………….4

**S2. Gene prediction and annotation**...........................................................................5

S2.1 Repetitive elements identification.................................................................5

S2.2 RNA-Seq of different tissues.........................................................................5

S2.3 RNA-Seq mapping and transcript reconstruction..........................................6

S2.4 High confidence (HC) and Medium Confidence (MC) *Phalaenopsis* gene set.................................................................................................................6

S2.5 Analysis of non-coding RNAs.......................................................................6

**S3. *Phalaenopsis* gene family analysis**........................................................................7

S3.1 Detection of gene families from the *Phalaenopsis* genome using OrthoMCL..................................................................................................7

S3.2 Comparison of the *Phalaenopsis*, *Oryza*, *Arabidopsis* and *Vitis* gene families.......................................................................................................7

S3.3 Transcription factor in *Phalaenopsis*.............................................................7

S3.3.1 TCP genes...........................................................................................7

S3.3.2 WRKY genes......................................................................................8

**S4. miRNA analysis**......................................................................................................8

S4.1 Small RNA library development and sequencing..........................................8

S4.2 miRNA gene and target prediction.................................................................9

S4.3 GO and Pfam analysis of the target genes.....................................................9

S4.4 miRNA and target gene analysis....................................................................9

**S5. Regulation of *Phalaenopsis* floral organ development and flowering time**....10

S5.1 Genes involved in floral organ development……………………………...10

S5.2 Genes involved in flowering time regulation..............................................11

**S6. Molecular marker development**………………………………………………..12

S6.1 Simple sequence repeat (SSR) analysis.......................................................12

S6.2 Single nucleotide polymorphism (SNP) analysis.........................................13

**SI FIGURES**………………………………………………………………………...14

**SI TABLES**…………………………………………………………………………..29

**SI Reference**………………………………………………………………………....40

**Supplementary Note**

**S1. Genome sequencing and assembly**

**S1.1 Whole-genome shotgun sequencing using Illumina technology**

The *Phalaenopsis* Brother Spring Dancer ‘KHM190’ orchid hybrid (Fig. S1a) was obtained from I-Hsin Biotechnology (Chiayi, Taiwan) and grown in a fan-and-pad greenhouse at National Pingtung University of Science and Technology (Pingtung, Taiwan), under natural daylight and at controlled temperatures of 27 to 30°C. Total DNA was extracted from *Phalaenopsis* leaves using a standard CTAB method (Doyle & Doyle 1987).

**S1.2 Library construction, sequencing and quality control**

To sequence the *Phalaenopsis* genome, we applied a whole-genome shotgun strategy and next-generation sequencing technologies using the Illumina HiSeq 2000 platform. Illumina short-insert paired-end (insert size: 250 bp) and large-insert mate-pair (3, 5, and 8 kb) libraries were prepared following the manufacturer’s instructions. In total, we generated approximately 300.5 Gb of sequences, and 278.89 Gb were retained for assembly after performing quality trimming using CLC Genomic Workbench 5.5 (http://www.clcbio.com/) to filter out low-quality reads (Table S1).

**S1.3 Estimation of genome size through K-mer analysis**

We adopted a method based on the K-mer distribution to estimate genome size. We used higher-quality reads (215.9 Gb) from short-insert size libraries (250 bp) to obtain more accurate estimations. A K-mer refers to an artificial sequence division of K nucleotides. Under this definition, a raw sequence read with L bp contains (L – K + 1) K-mers. The frequency of each K-mer was calculated from the genomic reads, and the K-mer frequencies followed a Poisson distribution for a deep-sequenced genome. Thus, the genome size, G, is calculated as G = Knum / Kdepth, where Knum is the total number of K-mers, and Kdepth is the highest peak detected. K was set to 17 in our project based on our empirical analysis. In this work, K was 17; Knum was 175,493,961,632; and Kdepth was 50. We therefore estimated the *Phalaenopsis* genome size to be 3.45 Gb (Fig. S2 and Table S2).

**S1.4 *De novo* assembly of the *Phalaenopsis* genome**

We performed whole-genome assembly using Velvet (<https://www.ebi.ac.uk/~zerbino/velvet/>) (Zerbino & Birney 2008) with K-mer 63. To fill the gaps inside the constructed scaffolds, we use Gapcloser to reduce the N ratio in the final assembly (<http://soap.genomics.org.cn/down/GapCloser_release_2011.tar.gz>) (Luo et al. 2012). The total contig length of the assembly reached 2.39 Gb (69.28% of 3.45 Gb), with an N50 length of 1.49 kb (longest, 50.94 kb), and the genome assembly was 3.1 Gb, with a scaffold N50 length of 100.94 kb (longest, 1.4 Mb) (Fig. S3 and Table S3). Scaffolds with lengths greater than 10 kb accounted for more than 74.8% of the assembly (Table S4).

**S1.5 Assembly evaluation**

The assembled genome of *Phalaenopsis* was validated using 8,188 Sanger-derived ESTs for *Phalaenopsis* downloaded from NCBI and was aligned to the assembly using BLAT (Kent 2002) with the default parameters. As a result, 7,701 genes (95% identity and over 50% coverage) were matched to the *de novo* *Phalaenopsis* assembly. The validation procedure confirmed the presence of 6,928 genes in the *Phalaenopsis* genome assembled with stringent parameters (95% identity and 90% coverage) (Table S5). Thus, the draft sequences represent a considerable portion of the *Phalaenopsis* genome, with high quality and coverage.

**S1.6 GC comparison**

Using 500-bp non-overlapping sliding windows, we calculated the GC contents of four species (*Phalaenopsis*, *Arabidopsis thaliana* (2000), *Oryza sativa* japonica (2005), and *Vitis vinifera* (Jaillon et al. 2007)). All four species showed peaks of GC content between 0.3 and 0.4. The GC content of the *Phalaenopsis* genome was 34.6%, which was similar to the GC contents of *A. thaliana* (38%), *O. sativa* japonica (38.3%) and *V. vinifera* (36%) (Fig. S4a). In addition, the data revealed the relationship between the sequencing depth and GC content. Nearly all regions with a GC content between 20% and 60% presented a sequencing depth of > 20x coverage (Fig. S4b).

**S.1.7 Identification of differentially expressed transcripts**

To evaluate the expression of raw transcript, we first mapped the trimmed reads to raw transcript sequence using gapped alignment mode of the program Bowtie 2.2.1.0 (Langmead & Salzberg 2012). After alignment, raw transcript expression was quantified with the software package eXpress 1.3.0 (Roberts & Pachter 2013). The value of read counts from eXpress would be the input of DESeq (Anders & Huber 2010), an R software package, was used to test for differential expression. Genes with differential expression of at least two-fold change at P≤0.05.

**S2. Gene prediction and annotation**

**S2.1 Identification of repetitive elements**

There are two main types of repeats in the genome, tandem repeats and transposable elements (TEs). We used Tandem Repeats Finder (Version 4.04) (Benson 1999) and Repbase (composed of many transposable elements, Version 15.01) (Jurka et al. 2005) to identify tandem repeats in the *Phalaenopsis* genome. We identified transposable elements in the genome at the protein and DNA levels. At the protein level, RepeatProteinMask (Smit et al. 1996-2010), an updated tool in the RepeatMasker package (Version 4.0.2), was employed to conduct RM-BlastX searches against the transposable element protein database. At the DNA level, RepeatMasker (Smit et al. 1996-2010) was applied, using a combined library of Viridiplantae lineage-specific TEs in Repbase (RELEASE 2013/04/22) and a *de novo* repeat sequence library of the *Phalaenopsis* genome defined with RepeatModeler (Version 1.0.7) (Smit et al. 1996-2010). The TE sequences were classified based on the reported system. Long terminal repeat (LTR) retrotransposons, mainly consisting of Gypsy-type (24.43%) and Copia-type (4.43%) LTRs, were predominant (Table S6). In addition, we aligned the classified TE families to the consensus sequences in the Repbase library. The distribution of transposable element divergence rates showed a peak at 17% (Fig. S5).

**S2.2 RNA-Seq of different tissues**

To generate a comprehensive view of the *Phalaenopsis* transcriptome, we applied the Illumina HiSeq 2000 system to perform high-throughput RNA-Seq analyses of four different types of samples: shoot tip tissues from shortened stems, floral organs (sepal, petal and labellum), leaves and protocorm-like bodies (PLBs). RNA was isolated from frozen orchid tissues via the TriSolution method (GeneMark, Taipei). The RNA solution was then treated with RNase-free DNase I (Promega, Taipei) to eliminate contaminating DNA. The quantity and quality of the RNA were evaluated using an Experion automated electrophoresis system (Bio-Rad). RNA samples with an RNA quality indicator (RQI) >8 were sent to Yourgene Bioscience on dry ice (New Taipei City, Taiwan) for mRNA purification and cDNA construction. The cDNA library for transcriptome sequencing was constructed with the Illumina TruSeq RNA sample prep kit. First- and second-strand cDNA was synthesised using reverse transcriptase (Clontech) with random hexamer primers and then subjected to end repair, A-tailing and adaptor ligation. Then, a total of 15 libraries were constructed with an insert size ranging from 200 to 300 bp (Table S7), and PCR amplification was performed for 15 cycles.

**S2.3 RNA-Seq mapping and transcript reconstruction**

To annotate transcriptionally active regions of the *Phalaenopsis* genome, RNAs from four different tissues were sequenced using Illumina transcriptome sequencing technology (Table S7). The 100-bp paired ends of the samples were pooled, and each sample dataset was aligned against the library-based repeat-masked assembly of *Phalaenopsis* using Bowtie2 (v2.1.0.0) (Langmead & Salzberg 2012) and TopHat (v2.0.8b) (Trapnell et al. 2009) with the default settings and the previously determined mean inner distance between mate pairs. We utilised TopHat to identify exon-intron splicing junctions and refine the alignment of the RNA-Seq reads to the genome. Cufflinks software (v2.1.1) (Pollier et al. 2013; Trapnell et al. 2012) was then employed to define a final set of predicted genes. Using the RNA-Seq approach, we predicted 54,659 gene loci and 76,370 spliced transcripts in the assembly (Dataset S1 and S2).

**S2.4 High-confidence (HC) and Medium-confidence (MC) *Phalaenopsis* gene set**

In total, 54,659 protein-coding loci were predicted. Of these loci, 41,153 were well supported (30~100% coverage) by either ESTs or NCBI proteins and were classified as high-confidence (HC) and medium-confidence (MC) genes. The HC and MC gene set comprised 41,153 genes predicted by Cufflinks (Pollier et al. 2013; Trapnell et al. 2012) (Dataset S3). Of the remaining genes, 13,506 were classified as low-confidence (LC); for these genes, the EST and/or protein alignment coverage was < 30%**.** The HC and MC gene sets were used to perform gene family analyses and various expression analyses.

**S2.5 Analysis of non-coding RNAs**

Noncoding RNAs, including rRNAs, tRNAs, snRNAs and snoRNAs, were predicted in the *Phalaenopsis* genome. To predict *Phalaenopsis* tRNA genes, we used tRNAscan-SE (v 1.23) (Lowe & Eddy 1997) with eukaryote parameters. We predicted 655 tRNA genes with an average length of 74.5 bp (Tables S8). The rRNA fragments were identified by aligning the rRNA template sequences (Rfam database release 11.0) (Burge et al. 2013; Gardner et al. 2009) against the *Phalaenopsis* genome using BLASTN with an e-value of 1e-5 and a cutoff identity of ≥85%. The snoRNA and snRNA genes were annotated using Bowtie 2 (Langmead & Salzberg 2012) software by searching against the Rfam database. We identified 562 rRNAs, 290 snoRNAs and 263 snRNAs in the *Phalaenopsis* genome (Table S8).

**S3. *Phalaenopsis* gene family analysis**

**S3.1 Detection of gene families from the *Phalaenopsis* genome using OrthoMCL**

We used OrthoMCL (v 1.4) (Chen et al. 2006; Li et al. 2003) to define gene family clusters for *Phalaenopsis*, *Oryza*, *Arabidopsis* and *Vitis* gene models. First, we employed Blastp with an E-value cutoff of 1e-5 and a minimum match length of 50% to compare protein sequences with a database containing the full protein datasets of the 4 selected species. To define the ortholog cluster structure, a Markov clustering algorithm (MCL) for the resulting similarity matrix (Szilagyi & Szilagyi 2013) was used to define the orthologous cluster structure, employing an inflation value (-I) of 1.5 (OrthoMCL default). Splice variants were removed from the dataset, and the longest predicted protein sequences were subsequently filtered for premature stop codons and incompatible sequences.

**S3.2 Comparison of the *Phalaenopsis*, *Oryza*, *Arabidopsis* and *Vitis* gene families**

A total of 142,785 sequences from *Phalaenopsis*, *Oryza*, *Arabidopsis* and *Vitis* were clustered into 23,420 gene families using OrthoMCL. A total of 8,532 clusters contained sequences from all four genomes. Among the 41,153 protein-coding sequences predicted for *Phalaenopsis*, 37,324 genes were clustered in a total of 15,885 families.

**S3.3 Transcription factors in *Phalaenopsis***

Transcription factors (TFs) are key regulators of the transcriptional expression of genes in biological processes. To identify TF families, we performed classification based on the rules of PlantTFDB v3.0 (Jin et al. 2014) (http://planttfdb.cbi.edu.cn/) for TF domain structure. In total, 3,309 predicted TFs were identified, including 56 families and representing 6.34% of the 41,153 predicted protein-coding loci. The most highly represented TF families were the bHLH (279 genes), AP2/EREBP (271 genes), NAC (224 genes), MYB-related (211 genes) and MYB (179 genes) families (Dataset S11 and S12). We performed a detailed phylogenetic analysis and identified different expression patterns of 3 well-known transcription factor families to provide highly focused views of gene family expansion and contraction in *Phalaenopsis*. An HMMER (v3.0) (Finn et al. 2011) search was also conducted for defined TCP and WRKY gene family sequences.

**3.3.1 TCP genes**

The TCP genes comprise a plant-speciﬁc transcription factor family with a basic helix-loop-helix structure that allows DNA binding and protein-protein interactions 38. The name TCP is derived from the founding members of the family: *Teosinte Branched 1* (*TB1*) from maize, the *Antirrhinum* gene *Cycloidea* (*CYC*), and two PCNA promoter-binding factors, *PCF1* and *PCF2*, from rice (Cubas 2004; Martin-Trillo & Cubas 2010). Members of the TCP family play crucial roles regulating the differentiation of shape and size in floral organs and leaves (Barkoulas et al. 2007; Cubas et al. 1999), vegetative branching patterns (Aguilar-Martinez et al. 2007; Doebley et al. 1997; Takeda et al. 2003), bilateral symmetry in several plant species and cell division (Cubas 2004).

In *Phalaenopsis*, 57 members of the TCP family were identified (Fig. S9). The trees were computed and drawn with ClustalW and MEGA5.1 (Dataset S11). The expression profile indicated that most TCP genes are widely expressed in diverse tissues (Dataset S11).

**3.3.2 WRKY genes**

Members of the plant WRKY gene family are ancient transcription factors that are involved in the regulation of various physiological processes, such as development and senescence, and in the plant response to many biotic and abiotic stresses45. This family contains at least one conserved DNA-binding domain with a highly conserved WRKYGQK heptapeptide sequence and a zinc finger motif (CX4-7CX22-23HXH/C or Cx7Cx23HXC) at the C-terminus (Rushton et al. 2010). In the *Phalaenopsis* genome sequence, a total of 164 genes were predicted to encode WRKY family proteins. Phylogenetic trees were computed and drawn with ClustalW and MEGA5.1 (Fig. S10). The expression patterns of WRKY genes were analysed in the *Phalaenopsis* global gene expression atlas in a variety of tissues using RNA-Seq analysis. All WRKY genes were expressed in at least one of the tissues (Dataset S11). In this study, we searched the *Phalaenopsis* genome sequence to identify the WRKY genes of *Phalaenopsis*. Detailed analysis, including gene classification, annotation, phylogenetic evaluation and expression profiling based on RNA-Seq data were performed on all members of the family. Our results provide a foundation for further comparative genomic analyses and functional studies on this important class of transcriptional regulators in *Phalaenopsis*.

**S4. miRNA analysis**

**S4.1 Small RNA library development and sequencing**

Total RNA was obtained from *Phalaenopsis* tissue samples, including the, shoot tip tissues of shortened stems, floral organs (sepal, petal and labellum), leaves and protocorm-like bodies (PLBs). We used 10 μg of total RNA as the initial input for library construction. Following 15% polyacrylamide denaturing gel electrophoresis, the small RNA fragments with lengths in the range of 16–32 nt were isolated from the gel and purified. Next, a *Phalaenopsis* small RNA library was prepared with the TruSeq Small RNA Sample Prep Kit (Illumina), using the Illumina TruSeq small RNA sample preparation protocol. Finally, the small RNA library was sequenced directly using the Illumina HiSeq 2000 platform at Yourgene Bioscience in Taiwan.

**S4.2 miRNA gene and target prediction**

The raw sequencing data were filtered with s Perl scripts to delete low-quality reads, adapters and contamination. The clean reads were aligned against plant repeat databases using Bowtie 2 software (Langmead & Salzberg 2012) to discard abundant non-coding RNAs (rRNA, tRNA, snRNA, and snoRNA) (http://rfam.sanger.ac.uk/ and <http://plantrepeats.plantbiology.msu.edu/>). The remaining unique filtered sequences were then compared with known mature and precursor miRNAs (pre-miRNAs) from other plant species deposited in miRBase 19 (Kozomara & Griffiths-Jones 2014)(http://www.mirbase.org/) using Bowtie software to search the conserved miRNAs. We used miRDeep2 (Friedlander et al. 2012) and INFERNAL (v 1.1) software (Nawrocki & Eddy 2013) to predict miRNA precursor sequences from the sequenced small RNAs.

The putative target sites of the miRNA candidates were identified by aligning the miRNA sequences with the assembled ESTs of *Phalaenopsis* using Bowtie software. The rules for target prediction were based on those of Allen et al. (2005) (Allen et al. 2005) and Schwab et al. (2005) (Schwab et al. 2005), in which mismatched bases were penalised according to their location in the alignment. To understand their biological function, these target genes were subjected to searches against the NCBI non-redundant database.

**S4.3 GO and Pfam analysis of target genes**

To better understand the functions of the miRNA targets, we performed GO analysis using the Blast2GO program (Conesa & Gotz 2008) and Pfam (Finn et al. 2014) based on Blastx hits against the NCBI Nr database, with an E-value threshold of less than 10-5.

**S4.4** **miRNA and target gene analysis**

We obtained 6,976,375 unique small RNA (sRNA) tags from 92,811,417 sRNA raw reads ranging from 18 to 27 bp (Dataset S6 and S7), among which the 24 nt category was the most abundant type of small RNA (34.59%) (Fig. S6). All of the conserved miRNA families showed a size distribution similar to their counterparts in *Arabidopsis* (Kasschau et al. 2007), *Oryza* (Jeong et al. 2011) and *Medicago* (Lelandais-Briere et al. 2009; Wang et al. 2011). The 650 miRNA sequences belong to 188 conserved miRNA families, with the number of members ranging from 1 to 23 (Dataset S8). Identification of miRNA targets is a prerequisite to understanding the functions of miRNAs. To identify potential targets of miRNAs, we screened *Phalaenopsis* transcriptomes in our database. As a result, we identified 1,644 potential target genes from 96 out of 188 miRNA families, and the representative targets for each miRNA family are listed in Dataset S9.

To better understand the functional roles of the predicted target genes in *Phalaenopsis*, we analysed the functional enrichment of all miRNA targets GO and Pfam analysis. The predicted miRNA targets showed enrichment in GO terms from the biological process, cellular component and molecular function categories. We identified 24 GO terms in the biological process category that showed strong enrichment in cellular and metabolic processes. In the cellular component category, the enriched GO terms included cell parts, organelles and organelle parts. In the molecular function category, the enriched GO terms included binding, catalytic activity and nucleic acid binding transcription factor activity (Fig. S7).

In addition, we investigated the assignments using homology searches against the Pfam database. A total 1,543 conserved protein domains with 603 variations were confirmed in the complete set of transcripts. PPR_2 (pfam13041) was first among these top domains, with a total of 129 hits. The second and third most frequent domains were PPR_1 (pfam12854) (64 hits) and PPR (pfam01535) (58 hits) (Dataset S10). We identified most transcription factor domains in *Phalaenopsis* transcriptomic sequences at E-values below 1e-4. Transcription factor genes are of particular importance because transcription factors may play a role in regulating the expression of other member genes. For example, AP2 and SBP family transcription factors, which are important in floral organ and lateral organ development and cell fate within the inflorescences of *Arabidopsis* (Chandler et al. 2007) and maize (Chuck et al. 2007), were predicted to be targets of mir-172 and mir-156, respectively (Dataset S9).

**S5. Regulation of *Phalaenopsis* floral organ development and flowering time**

**S5.1 Genes involved in floral organ development**

To investigate the potential mechanism underlying the variation in *Phalaenopsis* floral organ development, in the wild-type and peloric mutant of *Phalaenopsis* ‘KHM190’ (Fig. S11a and 11b), we evaluated the sepals, petals and labella of 0.2-cm buds through RNA-Seq analysis. The RNA-Seq data were mapped to the genomes using Bowtie and TopHat with the default parameters. We applied DEGseq software (Wang et al. 2010) to systematically screen for differentially expressed genes (DEGs) between the wild-type and peloric-mutant flower tissues (sepal, petal and labellum). Overall, a total of 1,838 genes were significantly differentially expressed between the peloric sepal (PS) and wild-type sepal (NS) libraries, 758 genes between the peloric petal (PP) and wild-type petal (NP) libraries and 1,147 genes between the peloric labellum (PL) and wild-type labellum (NL) libraries. To identify the most interesting candidates, we measured the levels of expression of 27 genes through real-time PCR analysis. Among these genes, *PhAGL6a*, *PhAGL6b* and *PhMADS4* stood out as the most interesting candidates. These genes were signiﬁcantly upregulated in the lip-like petals and lip-like sepals of the peloric mutant flowers. In addition, *PhAGL6b* was significantly downregulated in the labellum of the big lip mutant, with no change in the expression of *PhAGL6a* being observed (Huang et al. 2015). Furthermore, we cloned the full-length cDNA sequences of *PhAGL6a*, *PhAGL6b* and *PhMADS4* from the lip-like petal, lip-like sepal and big lip mutant of *Phalaenopsis*. Unexpectedly, we found that alternative splicing of *PhAGL6b* leads to the production of three different in-frame transcripts (*PhAGL6b-1*, *PhAGL6b-2* and *PhAGL6b-4*) and one frameshift transcript (*PhAGL6b-3*) only in the big lip mutant (Fig. S12).

**S5.2 Genes involved in the regulation of flowering time**

*Phalaenopsis* plants are usually grown at average daily temperatures of ≥28°C to promote leaf production and inhibit flower initiation, whereas a low-temperature regimen (25/20°C day/night) is used to induce flowering (Blanchard & Runkle 2006). Although some studies have demonstrated the importance of low-temperature requirements for flower initiation in *Phalaenopsis* (Chen et al. 2008), the underlying regulatory mechanism has yet to be elucidated. In this study, we aimed to identify the potential low-temperature transcriptional regulation of *Phalaenopsis* flowering time.

Thus, we performed a transcriptome analysis using the RNA-Seq method with mRNA from *Phalaenopsis* floral meristem tissues. The *P.* *aphrodite* orchid hybrid was obtained from Chainport Orchids (Pingtung, Taiwan) and grown in a fan-and-pad greenhouse at National Pingtung University of Science and Technology (Pingtung, Taiwan) under natural daylight and controlled temperatures of 27 to 30°C for 6 months. *Phalaenopsis* plants constituting the untreated group were subsequently grown at a constant high-temperature (BH) (30/27°C day/night) to inhibit flower initiation. Low-temperature (BL) treatment was carried out at 22/18°C (day/night) for 1 to 4 weeks. The RNA samples from the *Phalaenopsis* BL1~4 and BH shoot tip tissues of shortened stems described above were subjected to analysis on the Illumina HiSeq 2000 platform. We applied DEGseq software (Wang et al. 2010) to systematically screen for DEGs between the BL1~4 and BH groups. Furthermore, several criteria were applied to filter the refined list of DEGs in floral meristem tissues: a transcript should exhibit (1) ≥ 2 FPKM in at least one tissue and (2) a ≥ 2-fold-change compared with at least one of the other four tissues. Among the DEGs, 5,836, 6,415, 6,575 and 6,237 genes were upregulated, and 1,740, 1,894, 1,960 and 2,331 genes were downregulated, based on analysis of BL1/BH, BL2/BH, BL3/BH and BL4/BH, respectively (Fig. S13 and Dataset S13).

We focused our analysis of the *Phalaenopsis* floral meristem transcriptome on genes associated with flowering time regulation, an attribute that is extremely important for the transition to flowering. Therefore, we investigated the candidate genes whose annotation suggested a potential association with flowering time regulation. According to the annotation of unigenes, we obtained 86 genes related with flowering time. Some of them are listed in Dataset S14. These genes include photoperiod pathway genes such as GIGANTEA (GI), PHYTOCHROME INTERACTING FACTOR 3 (PIF3), LATE ELONGATED HYPOCOTYL (LHY), PHYTOCHROME A and B (PHYA, PHYB), and CONSTANS (CO); vernalization pathway genes related to VERNALIZATION (VRN), HETEROCHROMATIN1 (LHP1) and FRIGIDA (FRI); autonomous pathway genes related to FCA, FPA, FLOWERING LOCUS KH DOMAIN (FLK) and LUMINIDEPENDENS (LD); floral integrator pathway genes related to AP1, AP2, AGAMOUS (AGL), FLOWERING LOCUS T (FT), FRUITFULL (FUL) and LEAFY (LFY); and GA signalling pathway genes related to GIBBERELLIN BIOSYNTHESIS GENES, GIBBERELLIN RECEPTOR (GID), DELLA domain and GAMYB. Moreover, 122 MADS-box genes were uncovered (Dataset S11). These unigenes constitute important resources for future research on *Phalaenopsis* flowering time regulation.

**S6. Molecular marker development**

**S6.1 Simple sequence repeat (SSR) analysis**

SSRs are the most widely applied class of molecular markers used in genetic studies, with applications in many fields of genetics, including genetic conservation, population genetics and molecular breeding. SSRs in the *Phalaenopsis* genome were predicted using MIcroSAtelitte (MISA) (http://pgrc.ipk-gatersleben.de/misa/). The predicted SSRs were classified into five types according to their tandemly arranged copy number: di-, tri-, tetra-, penta- and hexa-nucleotide motifs repeated in tandem. We detected 532,285 SSRs in the *Phalaenopsis* genome. The statistics for the SSRs (di- up to hexamers) are shown in Table S9 and Fig. S14. In *Phalaenopsis*, dimers (79.71%) and trimers (15.75%) were the most abundant. We observed that SSRs were predominantly located in intergenic (84.33%) regions than in exonic (0.47%) and intronic (15.20%) regions in *Phalaenopsis* genome. To design SSR primers, were considered di-, tri-, tetra-, penta-, hexa- or compound repeat units, and 95,285 primer pairs were successfully designed that can be converted into genetic markers (Dataset S15).

**S6.2 Single nucleotide polymorphism (SNP) analysis**

SNPs are the most abundant type of molecular genetic marker in genomes, and numerous SNPs have been identified in many species (Feltus et al. 2004; Lam et al. 2010; Lu et al. 2012; Romay et al. 2013). To identify SNP markers in the *Phalaenopsis* genome, we re-sequenced the genome of *Phalaenopsis* *pulcherrima* ‘B8802’ which is a summer flowering species, and after filtering out low-quality reads, 30 Gb of the *Phalaenopsis* ‘B8802’ sequence were aligned to the *Phalaenopsis* ‘KHM190’ genome with scaffolds using CLC Genomics Workbench with the default settings. As a result, 75.2% of the reads were aligned to the *Phalaenopsis* ‘KHM190’ genome. Then, CLC Genomics Workbench was employed to call SNPs for this accession. We detected 691,532 SNP sites, including 20,654 homozygotes and 22,625 heterozygotes. Further analysis of the datasets showed that 9,364 SNPs were located in exons, 13,896 SNPs were located in introns and 20,019 SNPs were located in intergenic regions (Fig. S15 and Table S10 and Dataset S16).

**SI FIGURES**

**Figure S1.** ***Phalaenopsis* Brother Spring Dancer ‘KHM190’ (a) and *Phalaenopsis pulcherrima* ‘B8802’ (b and c) accessions used for genome sequencing.**


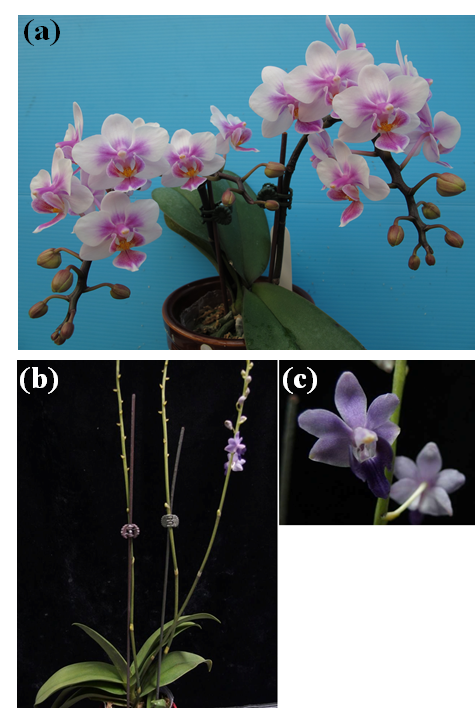


**Figure S2. Distribution of the 17-mer depth of the high-quality reads.**


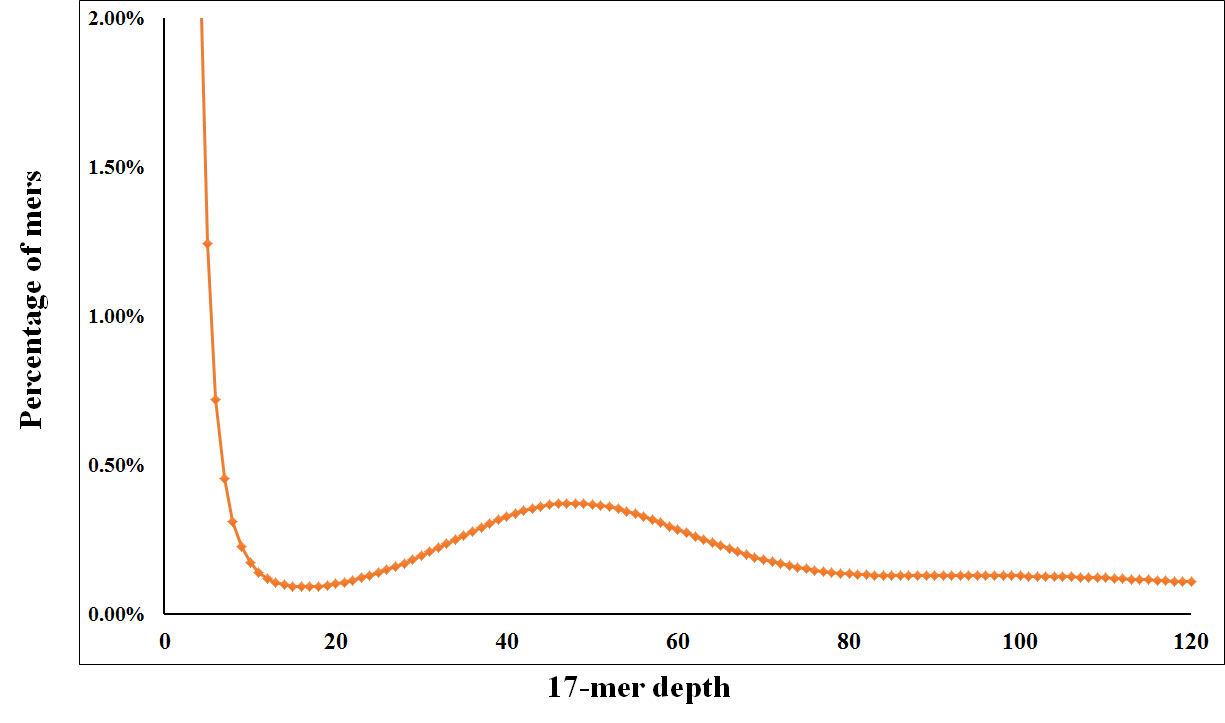


**Figure S3. Read-depth distribution in the *Phalaenopsis* genome assembly.**

**
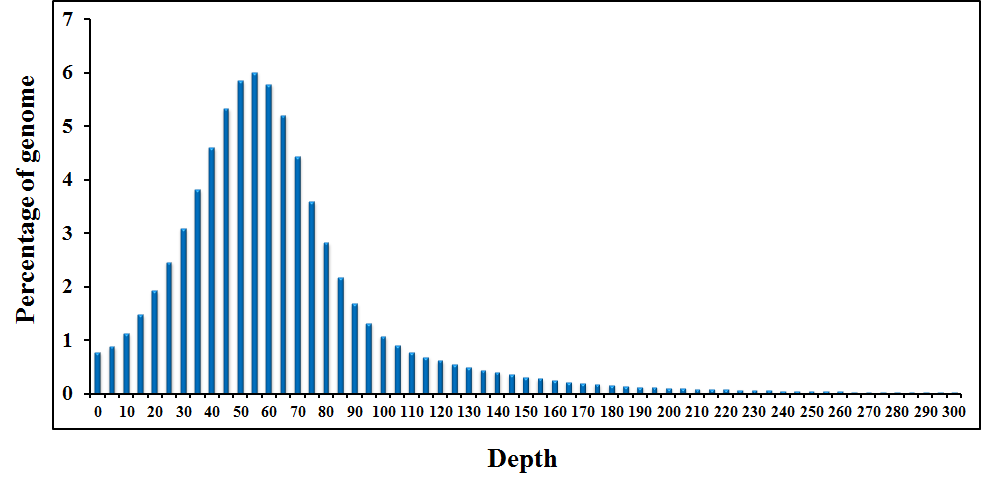
**

**Figure S4. Comparison of the GC content distribution between *Phalaenopsis* and three other plant species (a) and the GC content vs. the average sequencing depth in *Phalaenopsis* (b).**

**(a)**

**
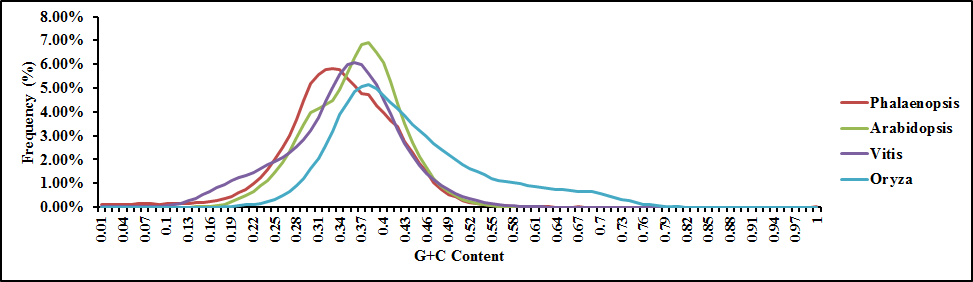
**

**(b)**

**
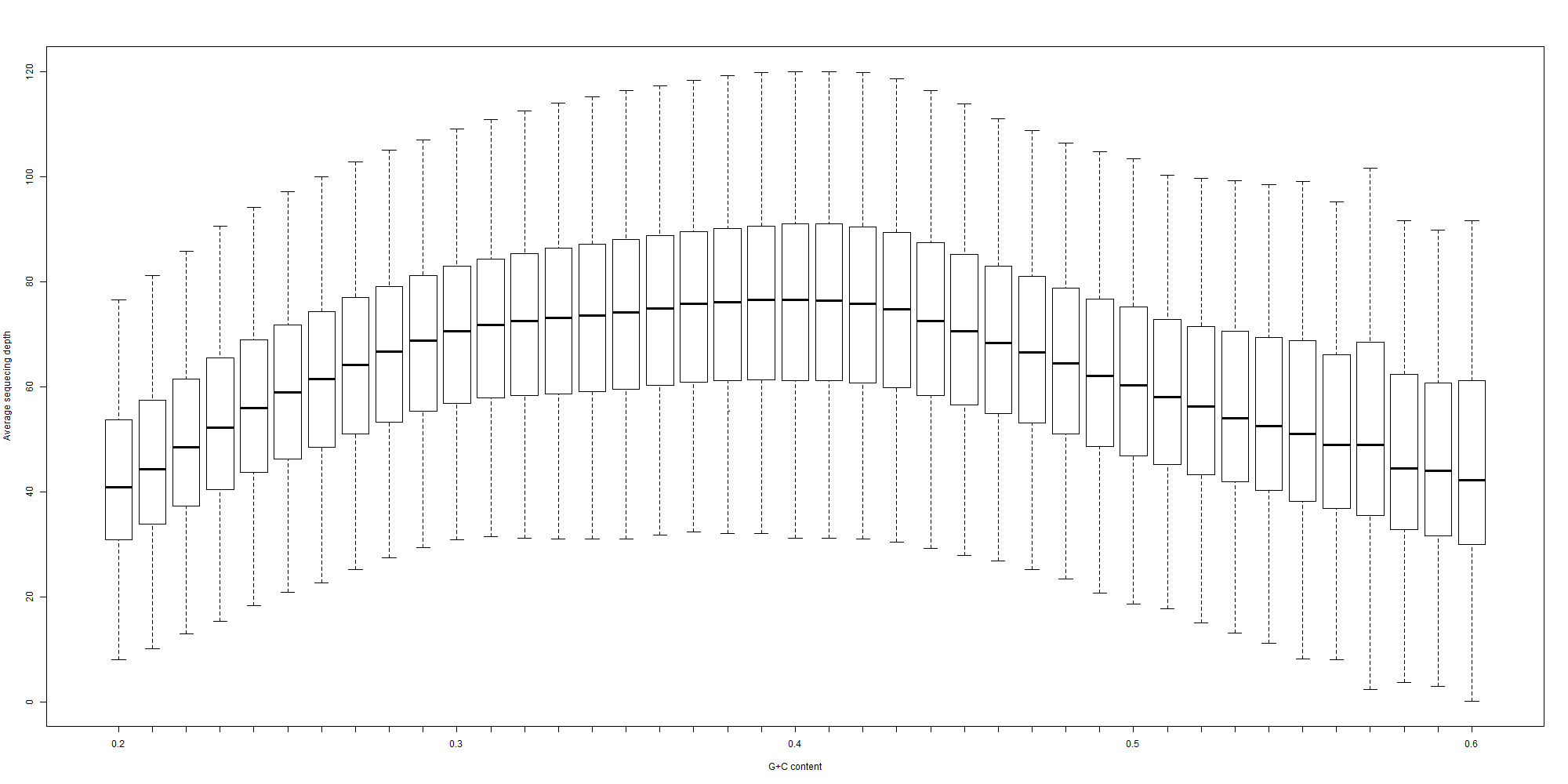
**

**Figure S5. Divergence distribution of the classified TE families in the *Phalaenopsis* genome. To analyse the divergence, different TE families were aligned onto the Repbase library. DNA: DNA elements; LINE: long interspersed nuclear elements; LTR: long terminal repeat transposable element; SINE: short interspersed nuclear elements.**

**
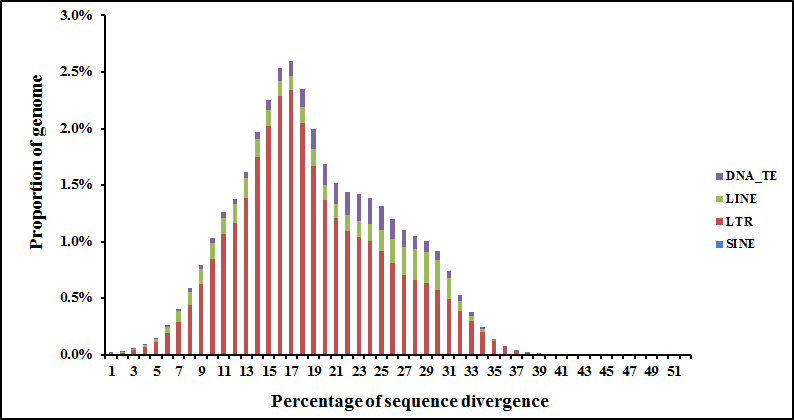
**

**Figure S6. Size distribution of small RNAs based on deep sequencing**

**
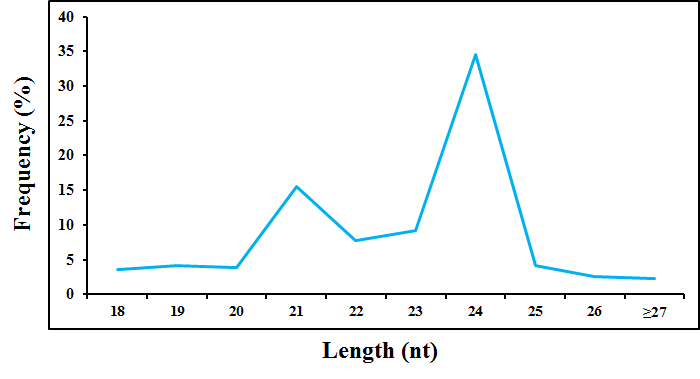
**

**Figure S7. GO analysis of miRNA target genes**

**
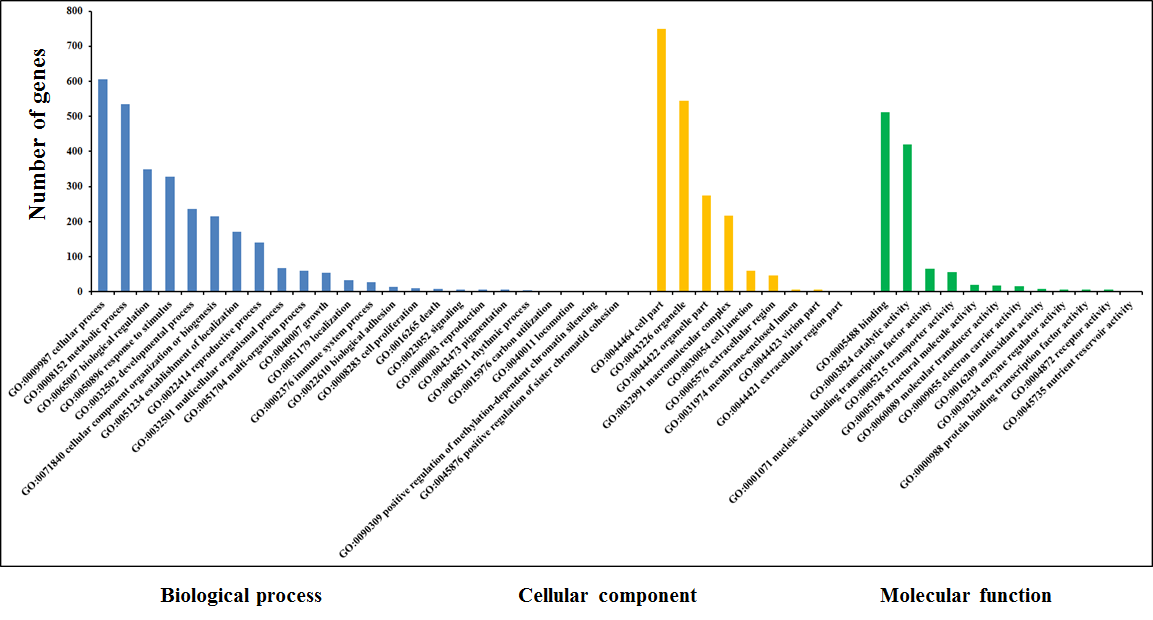
**

**Figure S8. Phylogenetic analysis of *PhMADS* genes in *Phalaenopsis*.**

**
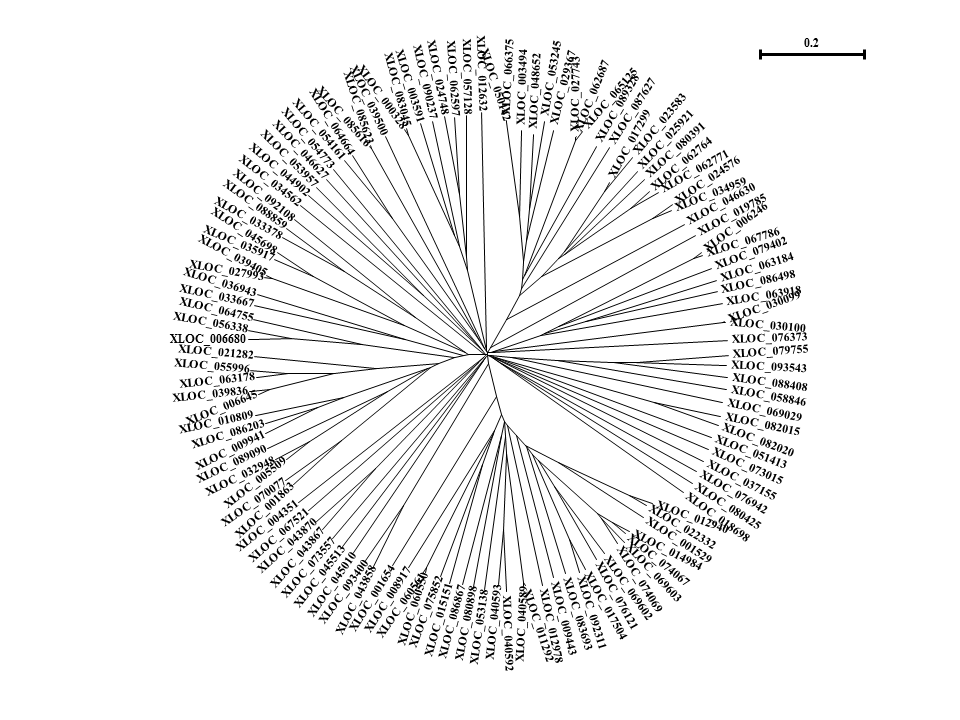
**

**Figure S9. Phylogenetic analysis of *PhTCP* genes in *Phalaenopsis*.**

**
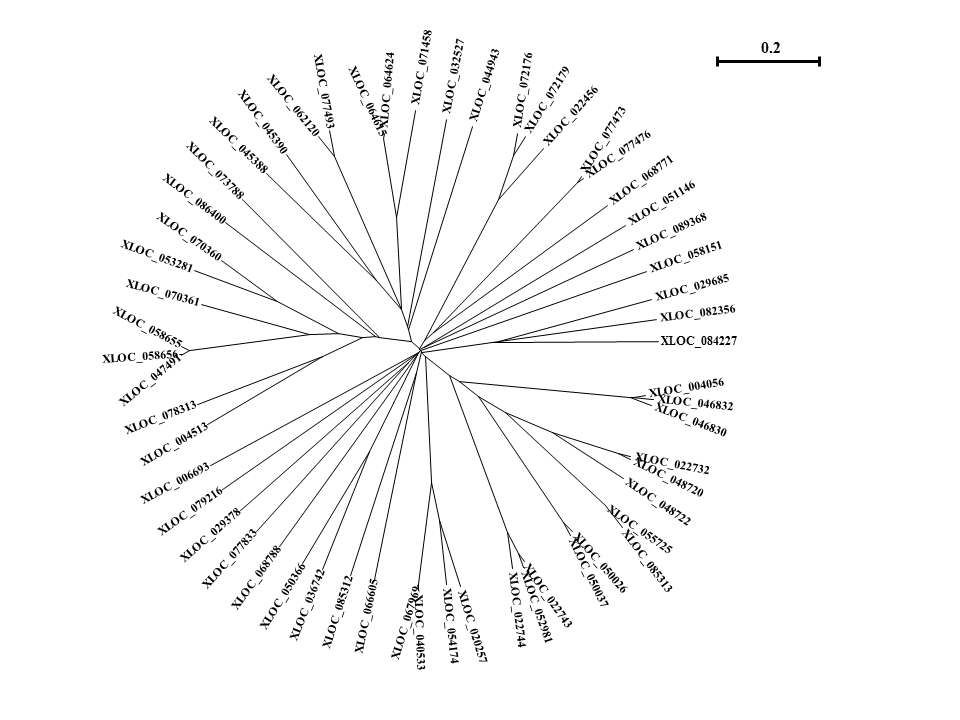
**

**Figure S10. Phylogenetic analysis of *PhWRKY* genes in *Phalaenopsis*.**

**
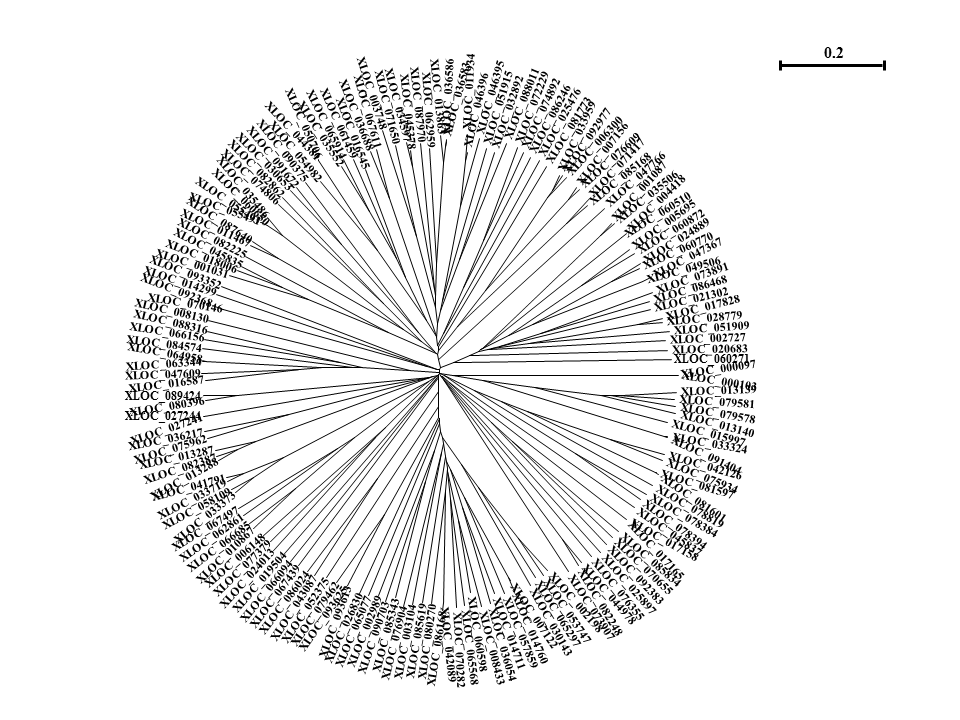
**

**Figure S11. Discovery of five splicing patterns of *PhAGL6b*. *PhAGL6b* represents the constitutive non-splicing form in wild-type labellum; *PhAGL6b-1*~*PhAGL6b-4* indicate alternative splicing forms in big lip mutants (a). Alignment of the nucleic acid sequences of alternatively spliced forms of *PhAGL6*** **(b).**

**(a)**


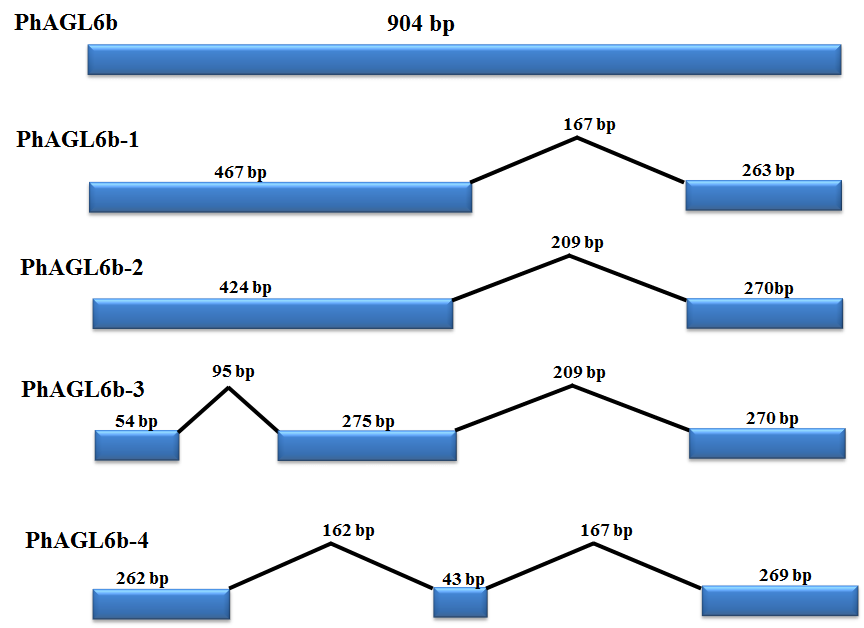


**(b)**

**
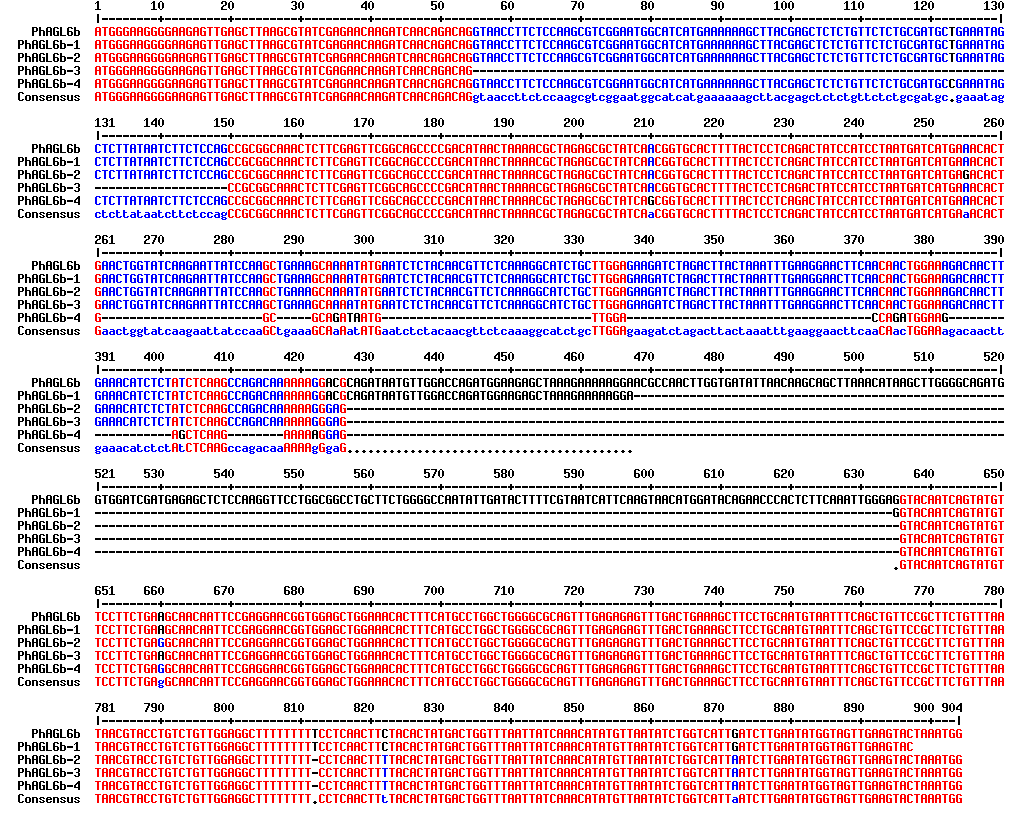
**

**Figure S12. Source tissues of Phalaenopsis orchids for transcriptome analysis. Flowers of the wild-type (a) and peloric mutant (b) of *Phalaenopsis* Brother Spring Dancer ‘KHM190’. Bar = 1 cm; Primary *Phalaenopsis* ‘KHM190’ PLB after excision and grown on induction medium for 0 week (c) and Primary *Phalaenopsis* ‘KHM190’ PLB after excision and grown on induction medium for 2 week (d). Bar = 05 cm; *Phalaenopsis aphrodite* wild type leaf (e) and *Phalaenopsis aphrodite* mutant with leaf intervein chlorosis (f). Bar = 5 cm; *Phalaenopsis aphrodite* with shoot tip tissues from shortened stems: constant high-temperature treatment was carried out at 30/27°C (day/night) (g) and low-temperature treatment was carried out at 22/18°C (day/night): 1 week (h), 2 week (i), 3 week (j), 4 week (k).**

**
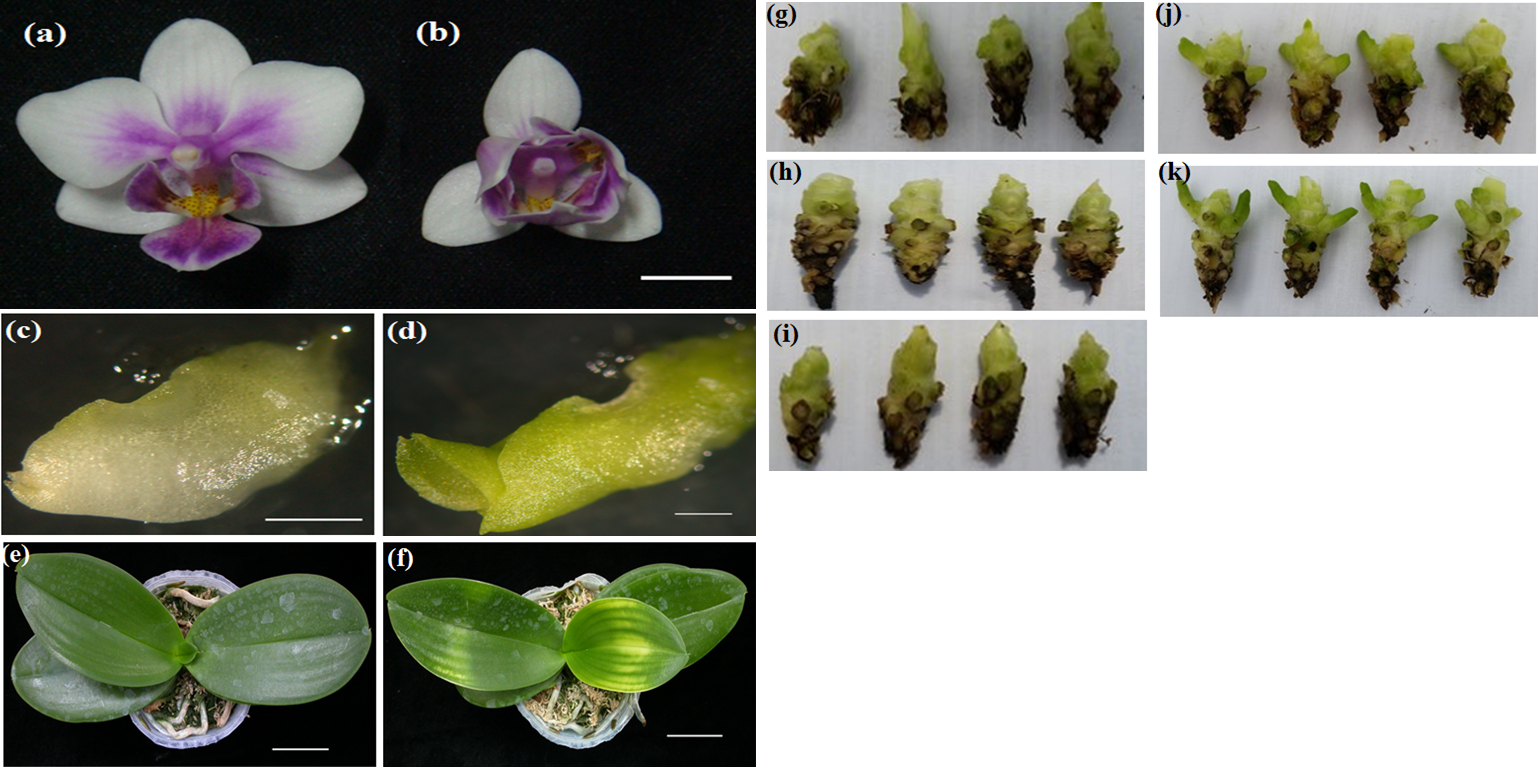
**

**Figure S13. Changes in gene expression profiles between constant high temperature (BH) and cool temperature (BL1~BL4; 1w to 4w) treatments. The numbers of up- and downregulated genes in BL1 and BH, BL2 and BH, BL3 and BH, and BL4 and BH. Five libraries are summarised**


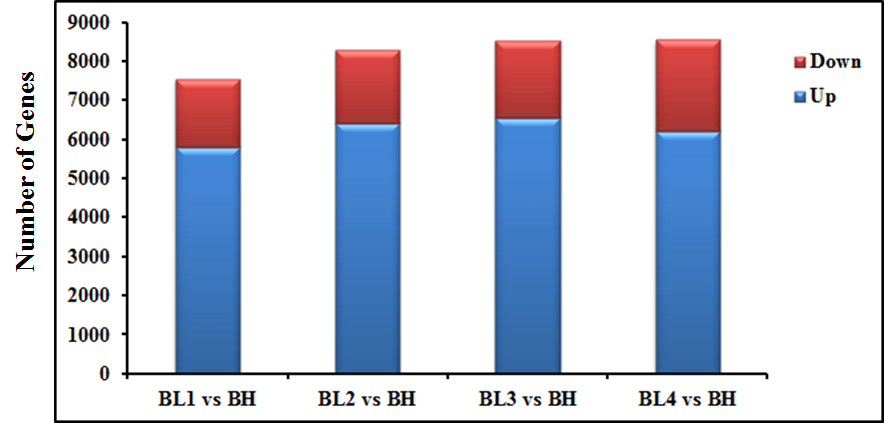


**Figure S14. Types and numbers of nucleotide motifs among the predicted SSR markers in *Phalaenopsis*. a, Di-; b, Tri-; c, Tetra-; d, Penta-; e, Hexa-nucleotide motifs.**

**
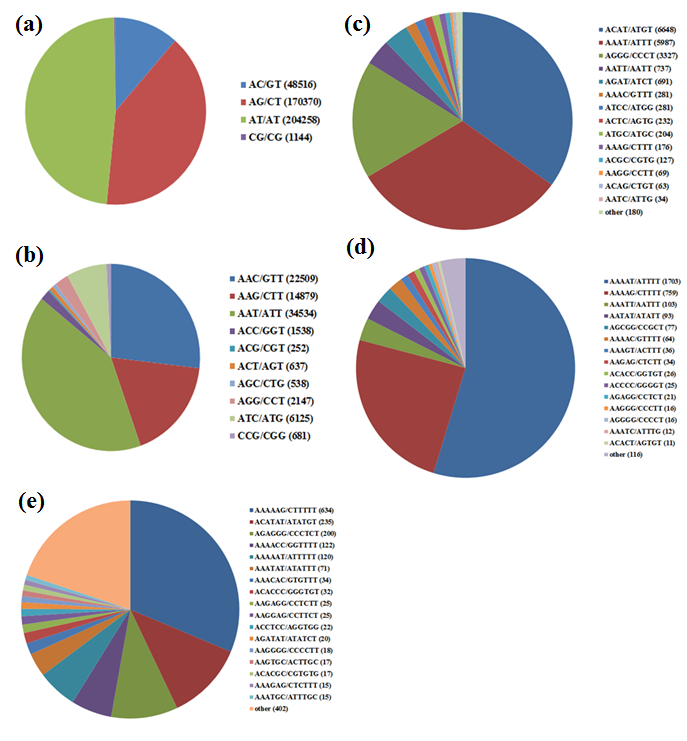
**

**Figure S15. (a) Base substitutions for SNPs between ‘KHM190’ and ‘B8802’. (b) Length distribution of indels. Small indels were identified between ‘KHM190’ and ‘B8802’.**

**(a)
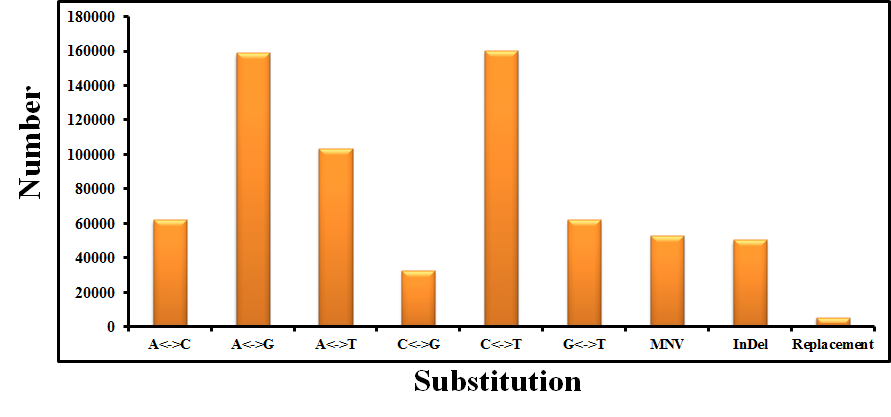
**

**(b)**

**
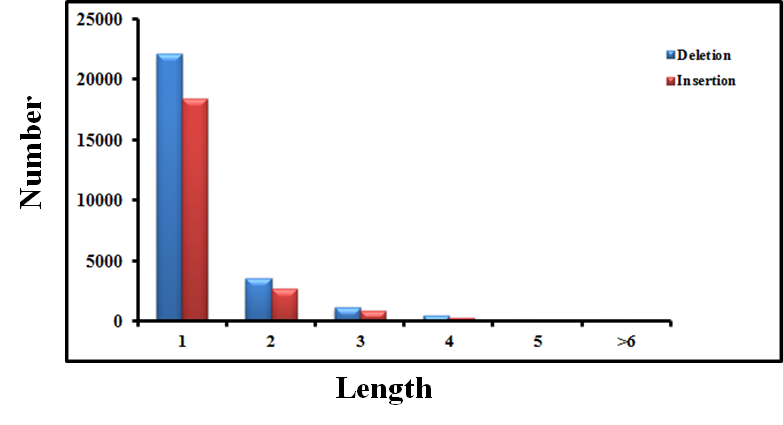
**

**SI TABLES**

**Table S1. Summary of sequencing data for the *Phalaenopsis* Brother Spring Dancer ‘KHM190’ genome.**

**Paired-end**

**insert size Raw reads Qualified reads**

**Total Reads Sequence Total Reads Sequence**

**data length coverage data length coverage**

**(Gb) (bp) (X) (Gb) (bp) (X)**

**250 bp 235.6 101 90.63 215.9 95 83.02**

**3 kb 7.65 101 2.94 7.2 96 2.76**

**5 kb 55.8 168 21.46 54.4 165 20.91**

**8 kb 1.4 101 0.55 1.4 99 0.54**

**Table S2. Statistics for the K-mer distribution.**

**Kmer Kmer number Kmer Genome Bases used Reads used Depth (X)**

**depth Size**

**17 175,493,961,632 50.0336 3,450,299,293 211,038,882,492 2,221,571,754 61.1654**

**Table S3. Summary of the *Phalaenopsis* genome assembly.**

**Contig Scaffold**

**Size (bp) Number Size (bp) Number**

**N90 239 1,963,018 492 304,925**

**N80 469 12,541,52 5,373 5,7336**

**N70 743 849,322 18,062 20,752**

**N60 1,075 581,348 54,618 10,961**

**N50 1,489 391,766 100,943 6,804**

**Longest 50,944 1,402,447**

**Total size 2,394,603,655 3,104,268,398**

**Total number (≥1 kb) 630,316 149,151**

**Total number (≥10 kb) 6,102 32,342**

**GC ratio (%) 34.6 30.7**

**Table S4. Distribution of scaffold length for the *Phalaenopsis* genome assembly.**

**Scaffold length (kb) Number Total length (bp) Average length (bp) Percentage (%)**

**>100 6,857 1,557,536,000 227,145 50.2**

**>10 32,342 2,321,440,805 71,777 74.8**

**>1 149,151 2,687,668,326 18,020 86.6**

**Table S5. Assessment of the sequence coverage of the *Phalaenopsis* genome assembly using ESTs.**

**EST length Number >50% of sequence >80% of sequence ≥ 90% of sequence**

**mapped by one mapped by one mapped by one**

**scaffold scaffold scaffold**

**Number Ratio (%) Number Ratio (%) Number Ratio (%)**

**All 8,188 7,701 94.05 7,610 92.94 6,928 84.61**

**>200 bp 7,669 7,294 95.11 7,204 93.93 6,535 85.21**

**>500 bp 5,418 5,162 95.27 5,082 93.80 4,673 86.25**

**Table S6. Summary of transposable elements in *Phalaenopsis*.**

**TE Classification Copies** **DNA Content (bp) DNA Content (%)**

**Class I: Retrotransposon 1,273,881 894,789,557 33.44**

**LTR-Retrotransposon** **1,071,162 777,601,961 29.05**

LTR/Gypsy 872,767 653,755,406 24.43

LTR/Copia 190,541 118,659,463 4.43

Other 7,854 5,187,092 0.19

**Non-LTR Retrotransposon 202,719 117,187,596 4.39**

LINE/L1 75,953 63,971,156 2.39

LINE/RTE-BovB 91,341 44,848,006 1.68

LINE/L2 28,098 5,785,445 0.22

LINE/I 7,327 2,582,989 0.10

**Class II: DNA Transposon 241,185 78,304,951 2.91**

CMC-EnSpm 64,545 22,518,411 0.84

hAT-Ac 91,599 22,317,836 0.83

PIF-Harbinger 44,475 18,675,443 0.70

MuLE-MuDR 14,171 6,916,377 0.25

hAT-Tag1 15,334 5,298,195 0.20

TcMar-Sagan 5,237 1,683,676 0.06

hAT-Tip100 5,824 895,013 0.03

Helitron 4,962 424,477 0.02

Satellite 9,082 8,577,798 0.32

Simple repeat 444,880 23,284,122 0.87

Low_complexity 81,701 5,120,206 0.19

**Unclassified**  **1,765,138 588,613,261 21.99**

**Total content** **3,820,829 1,598,926,178 59.74**

**Table S7. Transcriptome analysis of four organs of *Phalaenopsis* via RNA-Seq.**

**Organ**  **Usable data Transcripts Average length Maximum length Total size of transcripts**

**(Gb) (bp) (bp) (bp)**

**Shoot tip 35.2 56,609 914 19,384 51,769,072**

**Floral organs 32.5 40,192 1,081 17,075 43,464,697**

**Leaves 11.9 43,719 504 4,720 22,054,235**

**Protocorm-like body 9.9 61,736 653 5,042 40,324,120**

Shoot tip: Constant high temperature (BH) and a cool temperature (BL) (1 to 4 weeks)

Floral organs: sepal, petal and labellum tissues of both the ‘KH190’ wild-type and peloric mutant

Leaves: *Phalaenopsis aphrodite* wild type leaf and *Phalaenopsis aphrodite* mutant with leaf intervein chlorosis.

Protocorm-like body: *Phalaenopsis* Brother Spring Dancer ‘KHM190’ PLB after cutting and growth on induction medium for 0 week and Primary *Phalaenopsis* ‘KHM190’ PLB after cutting and growth on induction medium for 2 week.

**Table S8. Non-coding RNA genes in the *Phalaenopsis* genome.**

Type Copy Average length Total length

(bp) (bp)

miRNA 188 82.8 53,815

tRNA 655 74.5 48,802

rRNA

18S 51 1,127.9 57,525

28S 17 105.6 1,690

5.8S 168 153.9 25,859

5S 326 118.2 38,528

snoRNA

C/D-box 241 98.2 23,659

Other 49 87.6 4,290

snRNA 263 126.6 33,306

**Table S9. Summary of the types and numbers of simple sequence repeats (SSR) in the *Phalaenopsis* genome assembly.**

Motif Occurrence Most frequent type

Di- 424,288 AG/CT/GA/TC

Tri- 83,840 AAC/GTT/AAG/CTT/AAT

ATT/AGG/CCT/ATC/ATG

Tetra- 19,017 ACAT/ATGT/AAAT/ATTT

AGGG/CCCT

Penta- 3,112 AAAAT/ATTTT/AAAAG

CTTTT /AAATT/AATTT

Hexa- 2,028 AAAAAG/CTTTTT

ACATAT/ATATGT

AGAGGG/CCCTCT

AAAACC/GGTTTT

**Total 532,285**

**Table S10. Statistics for homozygous and heterozygous polymorphisms.**

**Sources Homozygous Heterozygous**

**SNP Indel SNP+Indel SNP Indel SNP+Indel**

**Gene region 9,946 566** **10,512 10,438 879** **11,317**

**Intergenic region 8,703 485** **9,188 8,832 752 9,584**

**Table S11. Primers used for cDNA cloning, RT-PCR analyses and real-time RT-PCR**

Primer Name Sequence

**For cloning and RT-PCR analysis**

PhAGL6-F: 5’ATGGGAAGGGGAAGAGTTGAGCTTAA3’

PhAGL6-R: 5’ TCAAACTGCGCCCCAGCCAGGCATGA3’

**For real-time RT-PCR**

PhAGL6qPCR-F 5’TAAGCTTGGGGCAGATGGTGG3’

PhAGL6qPCR-R 5’GTGGGTTCTGTATCCATGTTAC3’

PhAGL6-1qPCR-F 5’GAGCTAAAGAAAAAGGAGGTAC3’

PhAGL6-1qPCR-R 5’TCAAACTGCGCCCCAGCCAGGC3’

PhAGL6-3qPCR-F 5’AGATCAACAGACAGCCGCGGC3’

PhAGL6-3qPCR-R 5’ TTAGGATGGATAGTCTGAGGAG3’

PhAGL6-4qPCR-F 5’ATGATCATGAAACACTGGCGC3’

PhAGL6-4qPCR-R 5’TCAAACTGCGCCCCAGCCAGGC3’

**References**

2000. Analysis of the genome sequence of the flowering plant Arabidopsis thaliana. *Nature* 408:796-815. 10.1038/35048692

2005. The map-based sequence of the rice genome. *Nature* 436:793-800. 10.1038/nature03895

2008. The Gene Ontology project in 2008. *Nucleic Acids Res* 36:D440-444. 10.1093/nar/gkm883

Aguilar-Martinez JA, Poza-Carrion C, and Cubas P. 2007. Arabidopsis BRANCHED1 acts as an integrator of branching signals within axillary buds. *Plant Cell* 19:458-472. 10.1105/tpc.106.048934

Allen E, Xie Z, Gustafson AM, and Carrington JC. 2005. microRNA-directed phasing during trans-acting siRNA biogenesis in plants. *Cell* 121:207-221. 10.1016/j.cell.2005.04.004

Anders S, Huber W. 2010. Differential expression analysis for sequence count data. *Genome Biol* 11:R106. 10.1186/gb-2010-11-10-r106

Barkoulas M, Galinha C, Grigg SP, and Tsiantis M. 2007. From genes to shape: regulatory interactions in leaf development. *Curr Opin Plant Biol* 10:660-666. 10.1016/j.pbi.2007.07.012

Benson G. 1999. Tandem repeats finder: a program to analyze DNA sequences. *Nucleic Acids Res* 27:573-580.

Blanchard MG, and Runkle ES. 2006. Temperature during the day, but not during the night, controls flowering of Phalaenopsis orchids. *J Exp Bot* 57:4043-4049. 10.1093/jxb/erl176

Burge SW, Daub J, Eberhardt R, Tate J, Barquist L, Nawrocki EP, Eddy SR, Gardner PP, and Bateman A. 2013. Rfam 11.0: 10 years of RNA families. *Nucleic Acids Res* 41:D226-232. 10.1093/nar/gks1005

Chandler JW, Cole M, Flier A, Grewe B, and Werr W. 2007. The AP2 transcription factors DORNROSCHEN and DORNROSCHEN-LIKE redundantly control Arabidopsis embryo patterning via interaction with PHAVOLUTA. *Development* 134:1653-1662. 10.1242/dev.001016

Chen F, Mackey AJ, Stoeckert CJ, Jr., and Roos DS. 2006. OrthoMCL-DB: querying a comprehensive multi-species collection of ortholog groups. *Nucleic Acids Res* 34:D363-368. 10.1093/nar/gkj123

Chen WH, Tseng YC, Liu YC, Chuo CM, Chen PT, Tseng KM, Yeh YC, Ger MJ, and Wang HL. 2008. Cool-night temperature induces spike emergence and affects photosynthetic efficiency and metabolizable carbohydrate and organic acid pools in Phalaenopsis aphrodite. *Plant Cell Rep* 27:1667-1675. 10.1007/s00299-008-0591-0

Chuck G, Cigan AM, Saeteurn K, and Hake S. 2007. The heterochronic maize mutant Corngrass1 results from overexpression of a tandem microRNA. *Nat Genet* 39:544-549. 10.1038/ng2001

Conesa A, and Gotz S. 2008. Blast2GO: A comprehensive suite for functional analysis in plant genomics. *Int J Plant Genomics* 2008:619832. 10.1155/2008/619832

Cubas P. 2004. Floral zygomorphy, the recurring evolution of a successful trait. *Bioessays* 26:1175-1184. 10.1002/bies.20119

Cubas P, Vincent C, and Coen E. 1999. An epigenetic mutation responsible for natural variation in floral symmetry. *Nature* 401:157-161. 10.1038/43657

Doebley J, Stec A, and Hubbard L. 1997. The evolution of apical dominance in maize. *Nature* 386:485-488. 10.1038/386485a0

Doyle JJ, Doyle JL. 1987. A rapid DNA isolation procedure for small quantitiesof

fresh leaf tissue. *Phyt Bull* 19**:**11-15.

Feltus FA, Wan J, Schulze SR, Estill JC, Jiang N, and Paterson AH. 2004. An SNP resource for rice genetics and breeding based on subspecies indica and japonica genome alignments. *Genome Res* 14:1812-1819. 10.1101/gr.2479404

Finn RD, Bateman A, Clements J, Coggill P, Eberhardt RY, Eddy SR, Heger A, Hetherington K, Holm L, Mistry J, Sonnhammer EL, Tate J, and Punta M. 2014. Pfam: the protein families database. *Nucleic Acids Res* 42:D222-230. 10.1093/nar/gkt1223

Finn RD, Clements J, and Eddy SR. 2011. HMMER web server: interactive sequence similarity searching. *Nucleic Acids Res* 39:W29-37. 10.1093/nar/gkr367

Friedlander MR, Mackowiak SD, Li N, Chen W, and Rajewsky N. 2012. miRDeep2 accurately identifies known and hundreds of novel microRNA genes in seven animal clades. *Nucleic Acids Res* 40:37-52. 10.1093/nar/gkr688

Gardner PP, Daub J, Tate JG, Nawrocki EP, Kolbe DL, Lindgreen S, Wilkinson AC, Finn RD, Griffiths-Jones S, Eddy SR, and Bateman A. 2009. Rfam: updates to the RNA families database. *Nucleic Acids Res* 37:D136-140. 10.1093/nar/gkn766

Huang JZ, Lin CP, Cheng TC, Chang BC, Cheng SY, Chen YW, Lee CY, Chin SW, and Chen FC. 2015. A de novo floral transcriptome reveals clues into Phalaenopsis orchid flower development. *PLoS One* 10:e0123474. 10.1371/journal.pone.0123474

Jaillon O, Aury JM, Noel B, Policriti A, Clepet C, Casagrande A, Choisne N, Aubourg S, Vitulo N, Jubin C, Vezzi A, Legeai F, Hugueney P, Dasilva C, Horner D, Mica E, Jublot D, Poulain J, Bruyere C, Billault A, Segurens B, Gouyvenoux M, Ugarte E, Cattonaro F, Anthouard V, Vico V, Del Fabbro C, Alaux M, Di Gaspero G, Dumas V, Felice N, Paillard S, Juman I, Moroldo M, Scalabrin S, Canaguier A, Le Clainche I, Malacrida G, Durand E, Pesole G, Laucou V, Chatelet P, Merdinoglu D, Delledonne M, Pezzotti M, Lecharny A, Scarpelli C, Artiguenave F, Pe ME, Valle G, Morgante M, Caboche M, Adam-Blondon AF, Weissenbach J, Quetier F, and Wincker P. 2007. The grapevine genome sequence suggests ancestral hexaploidization in major angiosperm phyla. *Nature* 449:463-467. 10.1038/nature06148

Jeong DH, Park S, Zhai J, Gurazada SG, De Paoli E, Meyers BC, and Green PJ. 2011. Massive analysis of rice small RNAs: mechanistic implications of regulated microRNAs and variants for differential target RNA cleavage. *Plant Cell* 23:4185-4207. 10.1105/tpc.111.089045

Jin J, Zhang H, Kong L, Gao G, and Luo J. 2014. PlantTFDB 3.0: a portal for the functional and evolutionary study of plant transcription factors. *Nucleic Acids Res* 42:D1182-1187. 10.1093/nar/gkt1016

Jurka J, Kapitonov VV, Pavlicek A, Klonowski P, Kohany O, and Walichiewicz J. 2005. Repbase Update, a database of eukaryotic repetitive elements. *Cytogenet Genome Res* 110:462-467. 10.1159/000084979

Kasschau KD, Fahlgren N, Chapman EJ, Sullivan CM, Cumbie JS, Givan SA, and Carrington JC. 2007. Genome-wide profiling and analysis of Arabidopsis siRNAs. *PLoS Biol* 5:e57. 10.1371/journal.pbio.0050057

Kent WJ. 2002. BLAT--the BLAST-like alignment tool. *Genome Res* 12:656-664. 10.1101/gr.229202. Article published online before March 2002

Kozomara A, and Griffiths-Jones S. 2014. miRBase: annotating high confidence microRNAs using deep sequencing data. *Nucleic Acids Res* 42:D68-73. 10.1093/nar/gkt1181

Lam HM, Xu X, Liu X, Chen W, Yang G, Wong FL, Li MW, He W, Qin N, Wang B, Li J, Jian M, Wang J, Shao G, Sun SS, and Zhang G. 2010. Resequencing of 31 wild and cultivated soybean genomes identifies patterns of genetic diversity and selection. *Nat Genet* 42:1053-1059. 10.1038/ng.715

Langmead B, and Salzberg SL. 2012. Fast gapped-read alignment with Bowtie 2. *Nat Methods* 9:357-359. 10.1038/nmeth.1923

Lelandais-Briere C, Naya L, Sallet E, Calenge F, Frugier F, Hartmann C, Gouzy J, and Crespi M. 2009. Genome-wide Medicago truncatula small RNA analysis revealed novel microRNAs and isoforms differentially regulated in roots and nodules. *Plant Cell* 21:2780-2796. 10.1105/tpc.109.068130

Li L, Stoeckert CJ, Jr., and Roos DS. 2003. OrthoMCL: identification of ortholog groups for eukaryotic genomes. *Genome Res* 13:2178-2189. 10.1101/gr.1224503

Lowe TM, and Eddy SR. 1997. tRNAscan-SE: a program for improved detection of transfer RNA genes in genomic sequence. *Nucleic Acids Res* 25:955-964.

Lu P, Han X, Qi J, Yang J, Wijeratne AJ, Li T, and Ma H. 2012. Analysis of Arabidopsis genome-wide variations before and after meiosis and meiotic recombination by resequencing Landsberg erecta and all four products of a single meiosis. *Genome Res* 22:508-518. 10.1101/gr.127522.111

Luo R, Liu B, Xie Y, Li Z, Huang W, Yuan J, He G, Chen Y, Pan Q, Liu Y, Tang J, Wu G, Zhang H, Shi Y, Yu C, Wang B, Lu Y, Han C, Cheung DW, Yiu SM, Peng S, Xiaoqian Z, Liu G, Liao X, Li Y, Yang H, Wang J, and Lam TW. 2012. SOAPdenovo2: an empirically improved memory-efficient short-read de novo assembler. *Gigascience* 1:18. 10.1186/2047-217X-1-18

Martin-Trillo M, and Cubas P. 2010. TCP genes: a family snapshot ten years later. *Trends Plant Sci* 15:31-39. 10.1016/j.tplants.2009.11.003

Nawrocki EP, and Eddy SR. 2013. Infernal 1.1: 100-fold faster RNA homology searches. *Bioinformatics* 29:2933-2935. 10.1093/bioinformatics/btt509

Pollier J, Rombauts S, and Goossens A. 2013. Analysis of RNA-Seq data with TopHat and Cufflinks for genome-wide expression analysis of jasmonate-treated plants and plant cultures. *Methods Mol Biol* 1011:305-315. 10.1007/978-1-62703-414-2_24

Roberts A, Pachter L. 2013. Streaming fragment assignment for real-time analysis of sequencing experiments. *Nat Methods* 10: 71-73. 10.1038/nmeth.2251

Romay MC, Millard MJ, Glaubitz JC, Peiffer JA, Swarts KL, Casstevens TM, Elshire RJ, Acharya CB, Mitchell SE, Flint-Garcia SA, McMullen MD, Holland JB, Buckler ES, and Gardner CA. 2013. Comprehensive genotyping of the USA national maize inbred seed bank. *Genome Biol* 14:R55. 10.1186/gb-2013-14-6-r55

Rushton PJ, Somssich IE, Ringler P, and Shen QJ. 2010. WRKY transcription factors. *Trends Plant Sci* 15:247-258. 10.1016/j.tplants.2010.02.006

Schwab R, Palatnik JF, Riester M, Schommer C, Schmid M, and Weigel D. 2005. Specific effects of microRNAs on the plant transcriptome. *Dev Cell* 8:517-527. 10.1016/j.devcel.2005.01.018

Smit AFA, Hubley R, Green P. 1996-2010. RepeatMasker Open-3.0. [http://www.repeatmasker.org]

Szilagyi L, and Szilagyi SM. 2013. Efficient Markov clustering algorithm for protein sequence grouping. *Conf Proc IEEE Eng Med Biol Soc* 2013:639-642. 10.1109/EMBC.2013.6609581

Takeda T, Suwa Y, Suzuki M, Kitano H, Ueguchi-Tanaka M, Ashikari M, Matsuoka M, and Ueguchi C. 2003. The OsTB1 gene negatively regulates lateral branching in rice. *Plant J* 33:513-520.

Trapnell C, Pachter L, and Salzberg SL. 2009. TopHat: discovering splice junctions with RNA-Seq. *Bioinformatics* 25:1105-1111. 10.1093/bioinformatics/btp120

Trapnell C, Roberts A, Goff L, Pertea G, Kim D, Kelley DR, Pimentel H, Salzberg SL, Rinn JL, and Pachter L. 2012. Differential gene and transcript expression analysis of RNA-seq experiments with TopHat and Cufflinks. *Nat Protoc* 7:562-578. 10.1038/nprot.2012.016

Wang L, Feng Z, Wang X, and Zhang X. 2010. DEGseq: an R package for identifying differentially expressed genes from RNA-seq data. *Bioinformatics* 26:136-138. 10.1093/bioinformatics/btp612

Wang T, Chen L, Zhao M, Tian Q, and Zhang WH. 2011. Identification of drought-responsive microRNAs in Medicago truncatula by genome-wide high-throughput sequencing. *BMC Genomics* 12:367. 10.1186/1471-2164-12-367

Zerbino DR, and Birney E. 2008. Velvet: algorithms for de novo short read assembly using de Bruijn graphs. *Genome Res* 18:821-829. 10.1101/gr.074492.107
